# Wnt/β-catenin Signaling Inhibitors suppress the Tumor-initiating properties of a CD44^+^CD133^+^ subpopulation of Caco-2 cells

**DOI:** 10.7150/ijbs.58612

**Published:** 2021-04-12

**Authors:** Junghoon Kim, Kyeng-Won Choi, Jungwoon Lee, Jaeyoung Lee, Seonock Lee, Ruijing Sun, Jungho Kim

**Affiliations:** Laboratory of Molecular and Cellular Biology, Department of Life Science, Sogang University, Seoul 04107, Korea.

**Keywords:** Caco-2, Tumor-initiating cells, CD44, CD133, Tumorigenic potential, Wnt/β-catenin signaling inhibitor, XAV939, IWR-1

## Abstract

Tumor-initiating cells or cancer stem cells are a subset of cancer cells that have tumorigenic potential in human cancer. Although several markers have been proposed to distinguish tumor-initiating cells from colorectal cancer cells, little is known about how this subpopulation contributes to tumorigenesis. Here, we characterized a tumor-initiating cell subpopulation from Caco-2 colorectal cancer cells. Based on the findings that Caco-2 cell subpopulations express different cell surface markers, we were able to discriminate three main fractions, CD44^-^CD133^-^, CD44^-^CD133^+^, and CD44^+^CD133^+^ subsets, and characterized their biochemical and tumorigenic properties. Our results show that CD44^+^CD133^+^ cells possessed an unusual capacity to proliferate and could form tumors when transplanted into NSG mice. Additionally, primary tumors grown from CD44^+^CD133^+^ Caco-2 cells contained mixed populations of CD44^+^CD133^+^ and non-CD44^+^CD133^+^ Caco-2 cells, indicating that the full phenotypic heterogeneity of the parental Caco-2 cells was re-created. Notably, only the CD44^+^CD133^+^ subset of Caco-2-derived primary tumors had tumorigenic potential in NSG mice, and the tumor growth of CD44^+^CD133^+^ cells was faster in secondary xenografts than in primary transplants. Gene expression analysis revealed that the Wnt/β-catenin pathway was over-activated in CD44^+^CD133^+^ cells, and the growth and tumorigenic potential of this subpopulation were significantly suppressed by small-molecule Wnt/β-catenin signaling inhibitors. Our findings suggest that the CD44^+^CD133^+^ subpopulation from Caco-2 cells was highly enriched in tumorigenic cells and will be useful for investigating the mechanisms leading to human colorectal cancer development.

## Introduction

Colorectal cancer is one of the leading causes of cancer death around the world 1, 2. In general, human colorectal cancers originate from adenomatous polyps. These dysplastic, non-malignant precursor lesions can develop into malignant invasive colorectal cancers through multiple somatic cell mutations 3. Although several targeted therapies and other treatments have been tested in clinical trials to overcome the limitation of chemotherapeutic treatment, colorectal cancers are still challenging to cure and unfortunately many cases are fatal 4. A better understanding of the mechanisms underlying tumorigenesis is needed to reduce the incidence and mortality of human colorectal cancer.

Tumor initiation and maintenance are largely dependent on a small fraction of cancer cells, named tumor-initiating cells or cancer stem cells. Those types of cells exhibit indefinite self-renewal capacity, potential to induce tumorigenesis, and resistance to chemotherapeutic agents 5, 6. Conventional anti-cancer drugs target rapidly multiplying tumor cells; although these cells may respond transiently, the treatment ultimately fails to eradicate highly cancerous tumor-initiating cells, which are hard to kill using using standard chemotherapeutics and cause tumor recurrence 7-9. Tumor-initiating cells were initially identified in cases of acute myelogenous leukemia, in which only the immature CD34^+^CD38^-^ subset of leukemic cells, but not the CD34^+^CD38^+^ fraction, grow extensively, form tumors in mice, and repopulate the original tumor throughout several transplantations 10, 11. In addition, tumor-initiating cells have been isolated in several human cancers, including melanoma 12, 13, lung 14, 15, prostate 16, pancreatic 17, breast 18, glioma 19, liver 20, renal 21, skin squamous 22, esophageal 23, and colorectal 24, 25 cancers. Therefore, the elaborated characterization of tumor-initiating cells from human cancers may improve responses to treatment and lead to the identification of new treatments.

CD133 and/or CD44 proteins are important markers for a subset of human colorectal tumor-initiating cells 25-28. CD133 is a five-transmembrane glycoprotein expressed on the cell surface. It is also known as prominin-1 and is the human homolog of mouse prominin-1. Although its role remains unclear, it is widely accepted as a marker for tumor-initiating cells in human cancers 24, 25, 29-32. CD44 is also a family of cell surface glycoproteins. Notably, it is involved in tumor cell proliferation, adhesion, mobility, and metastasis 33, 34. Targeting CD44 with specific antibodies or siRNA has anti-tumor effects in nude mice 35, 36. Although human colorectal tumor-initiating cells are enriched for CD44^+^ 37, CD133^+^ 38, or CD44^+^CD133^+^ cells 26, 27, little is known about how these subpopulations contribute to tumorigenesis. Notably, the biological role and characteristics of the CD44^+^CD133^+^ subpopulation in human colorectal cancer remain incompletely understood.

To elucidate the pathways that regulate tumor-initiating cell growth and survival of Caco-2 cells, we isolated and characterized CD44^-^CD133^-^, CD44^-^CD133^+^, and CD44^+^CD133^+^ subpopulations. We found that the CD44^+^CD133^+^ fraction of Caco-2 cells is substantially enriched in tumor-initiating-like cells, which can be defined by functional analysis. CD44^+^CD133^+^ cells were larger and more mitotically active than CD44^-^CD133^-^ and CD44^-^CD133^+^ cells. Additionally, Caco-2 cells with this phenotypic characteristic formed tumors in immunodeficient NSG mice, whereas the subpopulations with alternative phenotypes failed to show tumorigenic potentials. Primary tumors grown from CD44^+^CD133^+^ Caco-2 cells contain mixed populations of both CD44^+^CD133^+^ and non-CD44^+^CD133^+^ Caco-2 cells, indicating re-creation of the full phenotypic heterogeneity of the parental Caco-2 cells. Notably, the Wnt/β-catenin pathway was more highly activated in CD44^+^CD133^+^ cells than in CD44^-^CD133^+^ cells. The expression of genes involved in the Wnt/β-catenin pathway was about 2.5- to 7-fold higher in the CD44^+^CD133^+^ subpopulation than in the CD44^-^CD133^+^ subpopulation, suggesting that the canonical Wnt/β-catenin pathway is over-activated in these cells. Furthermore, treatment with small-molecule Wnt/β-catenin signaling inhibitors significantly suppressed CD44^+^CD133^+^ cell proliferation and colony formation *in vitro* and tumorigenic capacity *in vivo*. These findings may thus provide insights to design more effective therapeutic strategies to eliminate human colorectal tumor-initiating cells.

## Materials and Methods

### Cell culture

Caco-2, HCT116, HT29, SW480, and DLD1 human colorectal cancer cell lines were purchased from the ATCC (American Type Culture Collection) and grown in MEM/EBSS (Minimal Essential Medium with Earle's Balanced Salts, Hyclone) supplemented with 10% heat-inactivated FBS (fetal bovine serum; Sigma-Aldrich), glutamine (GIBCO), and 1% penicillin-streptomycin (GIBCO) in a humidified 37 °C incubator (Thermo Fisher Scientific) with 5% CO_2_.

### FACS (fluorescence-activated cell sorting)

Standard cell surface flow cytometry was used to characterize Caco-2, HCT116, HT29, SW480, and DLD1 cells with FITC (fluorescein-5-isothiocyanate)-conjugated mouse anti-human CD44 (G44-26, BD Biosciences) and PE (phycoerythrin)-conjugated mouse anti-human CD133 (AC133, Miltenyi Biotech) antibodies. Cell sorting was performed using a FACSVantage SE flow cytometer (Becton Dickinson) with anti-CD44 and anti-CD133 antibodies. Data were analyzed with CellQuest software (Becton Dickinson).

### Cell growth curve

Equal numbers of CD44^-^CD133^-^, CD44^-^CD133^+^, and CD44^+^CD133^+^ subpopulations of Caco-2 cells (1 × 10^4^) were plated in duplicate in 35 mm cell culture dishes and cultured for 8 days. Cells were monitored at 2 day intervals after plating using an inverted phase-contrast microscope (IX71; Olympus). The total number of cells was counted in each plate using a hemocytometer, and the mean number was calculated.

### Cell cycle analysis

CD44^-^CD133^-^, CD44^-^CD133^+^, and CD44^+^CD133^+^ subpopulations of Caco-2 cells were collected 4 days after plating and rinsed two times in PBS (phosphate-buffered saline). The cells were fixed with 70% ethanol solution at -20 °C for 20 min and washed again twice with PBS. Fixed colorectal cancer cells were re-suspended in PBS containing 100 µg/ml RNase A, and RNAs in the cells were digested at 37 °C for 30 min. Finally, the cells were stained using propidium iodide solution (33 µg/ml propidium iodide, 10% NP-40) for 30 min and then used in flow cytometric analysis (FACSCalibur, Becton Dickinson).

### Microarray analysis

Total RNA was isolated from CD44^-^CD133^+^ and CD44^+^CD133^+^ Caco-2 cells with TRIzol reagent (Invitrogen). Quality control of RNA samples was assessed using a 2100 Bioanalyzer (Agilent Technologies), and then Cy3- (reference) and Cy5-labeled (sample) cRNAs were prepared. A whole genome human 44k microarray chip (Agilent Technologies) was hybridized with Cy3- and Cy5-labeled cRNAs using a gene expression hybridization kit (Agilent Technologies) following the manufacturer's instructions. The data for microarray analysis of CD44^-^CD133^+^ and CD44^+^CD133^+^ subpopulations of Caco-2 cells were deposited into the GEO database (https://www.ncbi.nlm.nih.gov/geo/) with the accession number GSE154750.

### Reverse transcription (RT)-PCR

Total RNAs were isolated from CD44^-^CD133^+^ and CD44^+^CD133^+^ Caco-2 cells with TRIzol solution (Invitrogen). A Superscript First-strand Synthesis System (Invitrogen) was used for cDNA synthesis, and then RT-PCR reactions for Wnt/β-catenin target genes were performed in triplicate. The primers used for amplification are listed in **Supplementary Table 1**.

### Quantitative real-time PCR

Quantitative real-time PCR was performed according to the manufacturer's instructions using the ABI 7500 Fast real-time PCR system (Applied Biosystems) with SYBR green master mix (Takara). β-actin mRNA was used for internal control. The primers used for real-time PCR are listed in **Supplementary Table 1**.

### Immunoblotting

Immunoblotting was performed using anti-phospho-β-catenin (Ser552; Cell Signaling Technology), anti-β-catenin (BD Transduction Laboratories), anti-Ki-67 (SP6; Abcam), anti-GAPDH (V-18; Santa Cruz Biotechnology), or anti-β-Actin (AbC-2002; AbClon) antibodies, and corresponding protein bands were detected by the Western Lightening system (PerkinElmer Life Sciences), according to the manufacturer's instructions.

### Serum starvation assay

Serum dependencies of CD44^+^CD133^+^ and ΔCD44^+^CD133^+^ (CD44^+^CD133^+^-negative; a subpopulation in which the CD44^+^CD133^+^ population was depleted) Caco-2 subpopulations were determined by comparing their growth rates in MEM/EBSS supplemented with 2% FBS. Each subpopulation was plated at a density of 2 × 10^4^ cells per well in a 12-well plate, and wells were maintained in 2% FBS for 9 days, with the addition of fresh media every 3 days.

### Crystal violet staining

Crystal violet fixing/staining reagent [0.05% (w/v) crystal violet (Sigma-Aldrich), 1% methanol (Sigma-Aldrich), 1% formaldehyde (Sigma-Aldrich), 1× PBS] was used to stain CD44^+^CD133^+^ and ΔCD44^+^CD133^+^ Caco-2 cells. Staining was performed for 20 min as described previously 39.

### Reporter gene assays

Freshly isolated CD44^-^CD133^+^ and CD44^+^CD133^+^ Caco-2 cells (1 × 10^5^ cells) were transfected transiently with pTOPFlash or pFOPFlash reporter plasmids using VivaMagic reagent (Vivagen Co., Ltd.) following the supplier's protocol. The Dual-Luciferase assay system (Promega) was used for luciferase assays, and transfection efficiencies were normalized using *Renilla* luciferase activity according to the manufacturer's instructions.

### Xenografts

Parental, CD44^+^CD133^+^, or ΔCD44^+^CD133^+^ Caco-2 cells (1 × 10^5^ cells) were suspended in a mixture (1:1 ratio) of media and Matrigel (Corning Life Sciences), and subcutaneously injected into NSG mice (Jackson Laboratory). NSG mice were anesthetized while cells were injected, and all mice were euthanized when the tumor measured 2 cm, or between 64 and 76 days post-transplantation.

To isolate cells from xenografts, freshly isolated primary tumors were made into a suspension of single cells using a tumor dissociation kit (Miltenyi Biotec). After isolating CD44^+^CD133^+^ and ΔCD44^+^CD133^+^ cells from the suspension using a FACSVantage SE flow cytometer (Becton Dickinson) with anti-CD44 and -CD133 antibodies, 1 × 10^5^ of each sorted cell population was then suspended in a mixture (1:1 ratio) of media and Matrigel, and was subcutaneously implanted into NSG mice.

To measure tumor suppression by the Wnt/β-catenin signaling inhibitor XAV939, CD44^+^CD133^+^ Caco-2 cells (1 × 10^5^ cells; cell suspension in 1:1 ratio of media and Matrigel) were injected into the flanks of 8-week-old NSG mice. When tumors became measurable (15-25 mm^3^), 20 mg/kg XAV939 per mouse or an equivalent volume of buffer alone was administered once every 3 days for 48 days. Mice that developed tumors were sacrificed 48 days after treatment began.

To determine the tumor volume, length (L, the greatest longitudinal diameter) and width (W, the greatest transverse diameter) were measured using an external calliper. Tumor volumes were calculated using the modified ellipsoidal formula [tumor volume = (L × W^2^)/2] as previously reported 39-41. All animal experiments in this study were approved by the committee for experimental animal research at Sogang University (IACUCSGU2020-14 and IACUCSGU2020-15) and performed following the animal experimentation guidelines of Sogang University, Seoul, Korea.

### Immunohistochemical staining

Formalin-fixed xenograft specimens were embedded into paraffin. Sections (4 μm thickness) were prepared using a RM2145 rotary microtome (Leica Microsystems). For immunohistochemistry analysis, sections were incubated with anti-CD44 (ab157107, Abcam) or anti-CD133 (ab19898, Abcam) primary antibodies overnight at 4 °C. The sections were then treated with HRP-conjugated secondary antibody; diaminobenzidine (ScyTek) was used as a chromogen, and sections were lightly counterstained with hematoxylin (ScyTek).

### Cell viability assay to calculate IC_50_ values

CD44^+^CD133^+^ Caco-2 cells (1 × 10^4^ per well) were plated into 12-well plates, and then varying concentrations of XAV939 (2.5-40 μM; Sigma-Aldrich) or IWR-1 (4.0-64 μM; Sigma-Aldrich) were added to the medium. The number of viable cells was measured after 6 days using an ADAM MC auto cell counter (Bulldog Bio). A nonlinear curve-fitting algorithm (SoftMax Pro software, Molecular Devices) was used to fit a sigmoidal curve to the average cell number plotted against XAV939 or IWR-1 concentration. The concentration of XAV939 or IWR-1 resulting in 50% maximal inhibition (IC_50_ value) of cell viability is presented.

### Statistical analysis

All data are shown as the mean ± standard deviation. The unpaired Student's t-test was used to compare two groups. Microsoft Excel software (Microsoft Corporation) was used for statistical analysis. *p* < 0.05 was considered statistically significant.

## Results

### CD44 and CD133 expression profile of representative colorectal cancer cell lines

We analyzed the expression profile of putative tumor-initiating cell markers, such as CD44 and CD133, in Caco-2, HCT116, HT29, SW480, and DLD1 colorectal cancer cell lines using flow cytometry (Table 1). The colorectal cancer cell lines that were studied expressed CD44 (CD44-positive, CD44^+^) and CD133 (CD133-positive, CD133^+^) at various frequencies. CD44 was the major marker expressed by HCT116 and HT29 cells. More than 80% of those cells expressed CD44 protein. However, about 77% of Caco-2 cells were CD44-negative (CD44^-^). CD133 was markedly expressed in Caco-2 (98%), HCT116 (83%), and HT29 (78%) cells, whereas SW480 and DLD1 showed low expression of CD133 (8% and 12%, respectively), indicating that the expression of CD44 was not strongly correlated with the expression of CD133 marker proteins in human colorectal cancer cell lines. These results also suggest that there is considerable heterogeneity among colorectal cancer cell lines in the expression of CD44 and CD133 markers.

**Figure 1:**
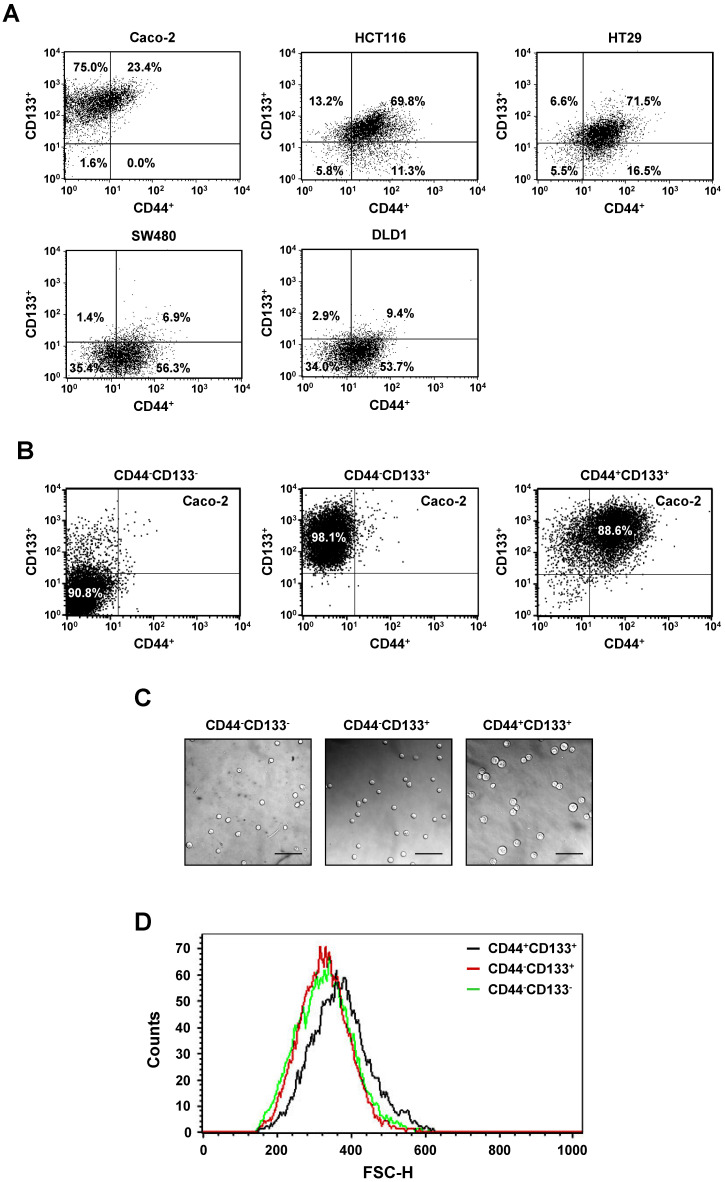
** Isolation and analysis of Caco-2 subpopulations expressing CD44 or/and CD133. (A)** Differences in the expression of CD44 and CD133 surface markers between colorectal cancer cells. Five colorectal cancer cell lines (Caco-2, HCT116, HT29, SW480, and DLD1) were cultured in MEM/EBSS (minimal essential medium with Earle's balanced salt solution) supplemented with 10% heat-inactivated FBS (fetal bovine serum), stained with specific monoclonal antibodies to CD44 (FITC-conjugated) and CD133 (PE-conjugated), and analyzed by flow cytometry. The results are expressed as the percentage of CD44^-^CD133^-^, CD44^+^CD133^-^, CD44^-^CD133^+^, or CD44^+^CD133^+^ cells in the total cell population. **(B)** Flow cytometric analyses of CD44^-^CD133^-^, CD44^-^CD133^+^, and CD44^+^CD133^+^ Caco-2 fractions to evaluate the efficiency of isolation. After FACS isolation, the percentage of CD44^-^CD133^-^, CD44^-^CD133^+^, and CD44^+^CD133^+^ cells within the total population increased to 90.8% ± 4.8%, 98.1% ± 1.1%, and 88.6% ± 4.0%, respectively. **(C)** Morphological characteristics of CD44^-^CD133^-^, CD44^-^CD133^+^, and CD44^+^CD133^+^ Caco-2 cells. CD44^-^CD133^-^, CD44^-^CD133^+^, and CD44^+^CD133^+^ cells (5 × 10^3^) were plated into 35 mm dishes, cultured for 24 hours, and examined using a confocal laser scanning microscope (LSM5 Pascal, Carl Zeiss Co., Ltd.; 200× original magnification). Scale bars, 100 μm. **(D)** Cell size distribution of CD44^-^CD133^-^, CD44^-^CD133^+^, and CD44^+^CD133^+^ Caco-2 cells. The *x*-axis of the plot represents forward scatter, indicating cell size, and the *y*-axis represents side scatter, indicating cell number. Curves denote CD44^-^CD133^-^ (green line), CD44^-^CD133^+^ (red line), and CD44^+^CD133^+^ (black line) cells.

We also investigated co-expression of CD44 and CD133 markers in colorectal cancer cell lines. Of the total population of Caco-2 cells, 23.4% were CD44^+^CD133^+^. SW480 and DLD1 cells contained only 6.9% and 9.4% CD44^+^CD133^+^ cells, respectively, in the total cell population. The population of HCT116 and HT29 cells each contained approximately 70% CD44^+^CD133^+^ cells, indicating that the frequencies of CD44^+^CD133^+^ subpopulations vary among colorectal cancer cell lines. Representative flow cytometric diagrams for CD44 and CD133 expression were presented in Figure 1A.

Among the five colorectal cancer cell lines used in Figure 1A, we selected Caco-2 cells for further experiments because they are a widely used human colorectal cancer cell line and are useful for colorectal carcinogenesis studies. Caco-2 cells were comprised of 1.6% CD44^-^CD133^-^ cells, 75.0% CD44^-^CD133^+^ cells, and 23.4% CD44^+^CD133^+^ cells; a CD44^+^CD133^-^ fraction was small or not observed (Figure 1A). To characterize the biochemical properties of the subpopulations of Caco-2 cells, CD44^-^CD133^-^, CD44^-^CD133^+^, and CD44^+^CD133^+^ cells were isolated from the total population of Caco-2 cells using flow cytometry. The isolated subpopulations were re-analyzed by flow cytometry to evaluate the efficiency of cell sorting, which showed that the proportions of CD44^-^CD133^-^, CD44^-^CD133^+^, and CD44^+^CD133^+^ cells in the “purified” fractions were 90.8%, 98.1%, and 88.6%, respectively (Figure 1B).

Microscopic examination revealed that the CD44^+^CD133^+^ subpopulation of Caco-2 cells were larger in diameter than the CD44^-^CD133^-^ or CD44^-^CD133^+^ cells (Figure 1C). Flow cytometry was used to characterize the size distribution of CD44^+^CD133^+^ cells. To measure cell size, 1 × 10^4^ CD44^-^CD133^-^, CD44^-^CD133^+^, or CD44^+^CD133^+^ Caco-2 cells were acquired and then analyzed using their forward scatter parameters. The size distribution of the CD44^+^CD133^+^ cells (Figure 1D, black line) shifted to the right of that of the CD44^-^CD133^-^ (green line) and CD44^-^CD133^+^ (red line) cells, indicating that CD44^+^CD133^+^ Caco-2 cells are larger than CD44^-^CD133^-^ and CD44^-^CD133^+^ cells (Figure 1D). However, CD44^-^CD133^-^ and CD44^-^CD133^+^ Caco-2 cells were the same or a similar size (Figure 1D, red and green lines).

### Growth characteristics of three subpopulations of Caco-2 cells

**Figure 2:**
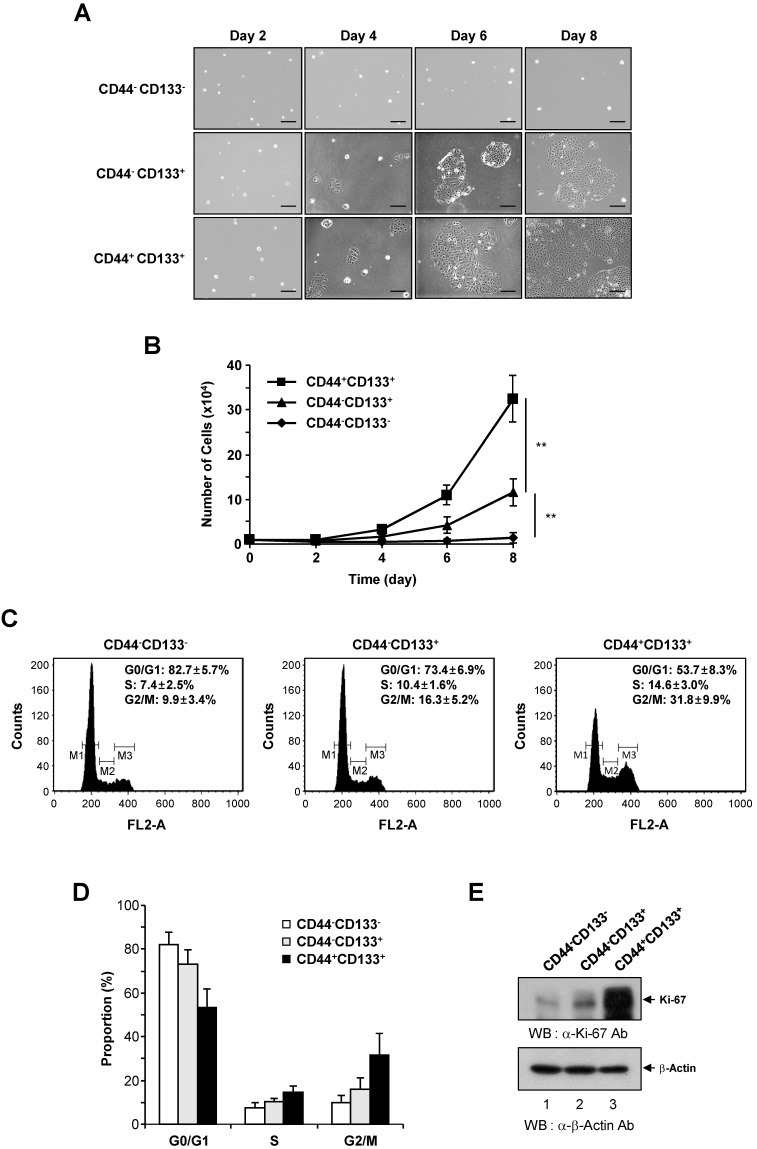
** Growth characteristics and cell cycle analysis of CD44^-^CD133^-^, CD44^-^CD133^+^, and CD44^+^CD133^+^ Caco-2 cells. (A)** Colony morphology of CD44^-^CD133^-^, CD44^-^CD133^+^, and CD44^+^CD133^+^ Caco-2 cells. CD44^-^CD133^-^, CD44^-^CD133^+^, and CD44^+^CD133^+^ cells (1 × 10^4^) were plated and cultured for 8 days. Cells were monitored at 2 day intervals under an inverted phase-contrast microscope (IX71; Olympus). Scale bars, 100 μm. **(B)** Growth curve of CD44^-^CD133^-^, CD44^-^CD133^+^, and CD44^+^CD133^+^ Caco-2 cells. Cells were seeded at 1 × 10^4^ cells per 35 mm dish and counted at 2 day intervals using a hemocytometer for a total of 8 days. Statistical significance was determined using unpaired Student's *t*-tests. **p < 0.01 versus control group. **(C)** Cell cycle distribution of CD44^-^CD133^-^, CD44^-^CD133^+^, and CD44^+^CD133^+^ Caco-2 cells. The DNA content of CD44^-^CD133^-^ (left panel), CD44^-^CD133^+^ (middle panel), and CD44^+^CD133^+^ (right panel) cells was measured by fluorescence after propidium iodide staining. M1, M2, and M3 represent the G0/G1, S, and G2/M phases, respectively. Values represent the mean ± SD for three independent experiments. **(D)** Proportion of CD44^-^CD133^-^, CD44^-^CD133^+^, and CD44^+^CD133^+^ Caco-2 cells at G0/G1, S, and G2/M phase. The percentage of cells at G0/G1, S, and G2/M are shown after flow cytometry. Values represent the mean ± SD for three independent experiments. **(E)** Upregulation of Ki-67 expression in CD44^+^CD133^+^ Caco-2 cells. The protein expression level of Ki-67 in CD44^-^CD133^-^, CD44^-^CD133^+^, and CD44^+^CD133^+^ cells were assessed by western blotting (WB) (upper panel). After resolving the extracts by SDS-PAGE, the samples were analyzed by immunoblotting using anti-Ki-67 (SP6; Abcam) or anti-β-Actin (AbC-2002; AbClon Inc.) antibodies (Ab). β-Actin served as a loading control. Three independent experiments gave similar results.

To determine differences in proliferation, CD44^-^CD133^-^, CD44^-^CD133^+^, and CD44^+^CD133^+^ subpopulations of Caco-2 cells were isolated by flow cytometry, and 1 × 10^4^ cells were plated into 35 mm dishes. Cells were monitored at 2 day intervals under an inverted phase-contrast microscope. CD44^+^CD133^+^ and CD44^-^CD133^+^ Caco-2 cells formed successive colonies and grew like normal unfractionated cells; the colony sizes of the CD44^+^CD133^+^ subpopulation were bigger than those of the CD44^-^CD133^+^ subpopulation (Figure 2A, second and third panels). Notably, CD44^-^CD133^-^ cells failed to form colonies (Figure 2A, top panels).

We further investigated the growth ability of CD44^-^CD133^-^, CD44^-^CD133^+^, and CD44^+^CD133^+^ Caco-2 cells. To compare proliferation potential, FACS-isolated subpopulations were cultured and counted at 2 day intervals for 8 days. The growth properties of CD44^+^CD133^+^ Caco-2 cells were markedly different from those of CD44^-^CD133^+^ and CD44^-^CD133^-^ Caco-2 cells. As shown in Figure 2B, CD44^+^CD133^+^ Caco-2 cells grew faster throughout the duration of the experiment than other subpopulations. While the microscopic morphology of the CD44^-^CD133^-^ and CD44^-^CD133^+^ Caco-2 cells was very similar (Figure 1C), the CD44^-^CD133^-^ subpopulation had limited proliferative capacity and finally lost their mitotic potential (Figure 2B).

To assess whether the CD44^+^CD133^+^ cell fraction was enriched for replication-competent cells, the cell cycle distribution of the CD44^-^CD133^-^, CD44^-^CD133^+^, and CD44^+^CD133^+^ subpopulations was investigated. DNA content was analyzed by propidium iodide staining and flow cytometry. The number of CD44^+^CD133^+^ cells in S-phase (Figure 2C, right panel) was higher than the number of CD44^-^CD133^+^ cells (Figure 2C, middle panel). A large proportion of CD44^-^CD133^-^ cells were in G0/G1 phase, accompanied by a low proportion in S-phase (Figure 2C, left panel), indicating a failure of cell cycle progression. The proportion of CD44^-^CD133^-^, CD44^-^CD133^+^, and CD44^+^CD133^+^ Caco-2 cells in G0/G1, S, and G2/M phases is presented in Figure 2D.

As a separate measure of proliferation, the expression of Ki-67, which is a novel marker of cell growth 42, was determined by western blotting of CD44^-^CD133^-^, CD44^-^CD133^+^, and CD44^+^CD133^+^ total cell extracts. Similar to the results of the growth assays (Figure 2B) and cell cycle analysis (Figure 2C), the expression of Ki-67 protein was significantly higher in CD44^+^CD133^+^ Caco-2 cells than in CD44^-^CD133^+^ and CD44^-^CD133^-^ cells (Figure 2E). Taken together, these data suggest that the Caco-2 subpopulations have different growth properties, with CD44^+^CD133^+^ cells being more mitotically active.

### The CD44^+^CD133^+^ subpopulation of Caco-2 cells has increased tumorigenic potential *in vivo*

**Figure 3:**
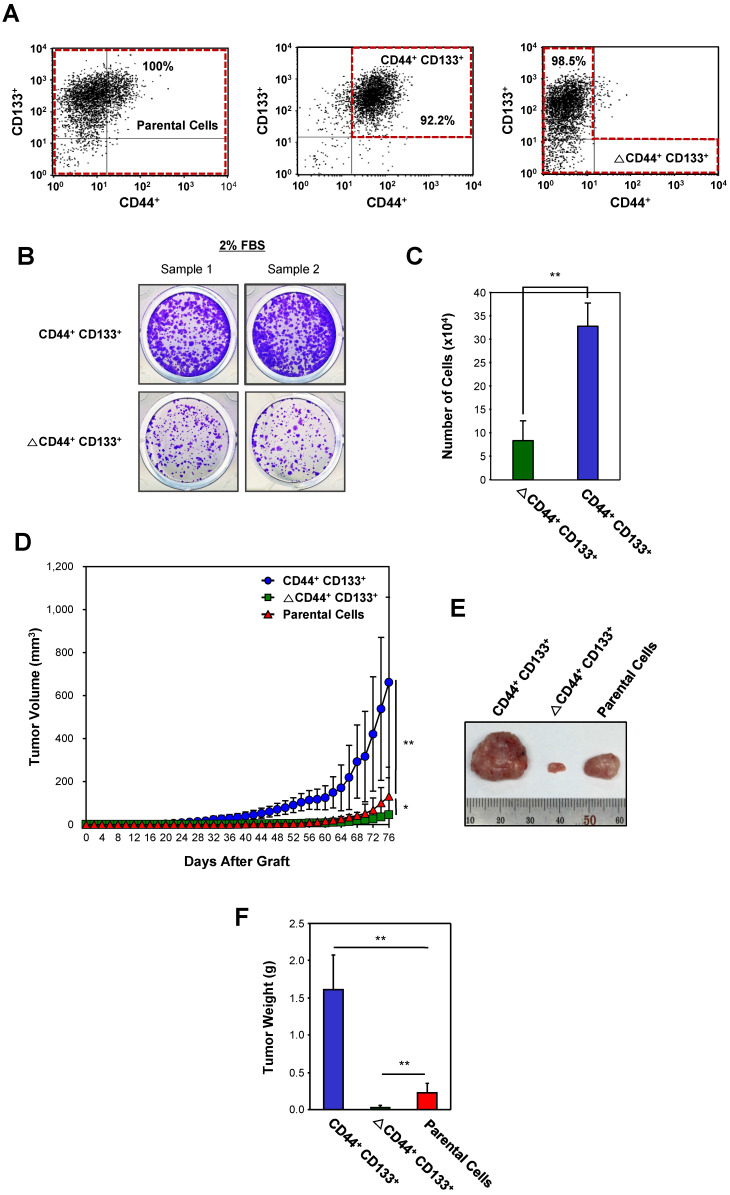
** Evaluation of the tumorigenicity of the CD44^+^CD133^+^ Caco-2 cells *in vivo*. (A)** Cytofluorimetric cell sorting of double-labeled parental (left panel), CD44^+^CD133^+^-positive (middle panel), and CD44^+^CD133^+^-negative (ΔCD44^+^CD133^+^, right panel) Caco-2 cells. Sorted cells were re-analyzed shortly after the initial sorting. Cell purities are presented as the percentage of selected cells in the sort fraction. **(B)** Analysis of serum requirements of Caco-2 cells expressing CD44 and CD133. CD44^+^CD133^+^ and ΔCD44^+^CD133^+^ cells were cultured in MEM/EBSS supplemented with 2% (v/v) FBS for 9 days and then stained with crystal violet to visualize cells. **(C)** Effect of serum starvation on CD44^+^CD133^+^ and ΔCD44^+^CD133^+^ Caco-2 cell proliferation. After serum starvation, total cell numbers were determined by counting at day 9. CD44^+^CD133^+^ (blue bar) and ΔCD44^+^CD133^+^ cells (green bar) were maintained by MEM/EBSS supplemented with 2% (v/v) FBS. Each bar represents the mean ± SD of three independent experiments. An unpaired Student's *t*-test was used to determine statistical significance. **p < 0.01 versus control group. **(D)** Growth of mouse xenografts generated after subcutaneous injection of unsorted (parental) or purified (CD44^+^CD133^+^ or ΔCD44^+^CD133^+^) Caco-2 cells. The parental, CD44^+^CD133^+^, or ΔCD44^+^CD133^+^ cells were sorted by flow cytometry and then subcutaneously injected into 8-week-old NSG mice (n = 7). Tumor development was observed at 2 day intervals for a total of 76 days. The volume of palpable tumors derived from parental (red triangles), CD44^+^CD133^+^ (blue circles), or ΔCD44^+^CD133^+^ (green squares) cells was measured and plotted as mean increases ± SD. An unpaired Student's *t*-test was used to determine statistical significance. *p < 0.05 and **p < 0.01 versus control group. **(E)** Photograph of tumor xenografts generated after subcutaneous injection of parental, CD44^+^CD133^+^, or ΔCD44^+^CD133^+^ Caco-2 cells. The representative tumor photograph was taken 76 days after injection. **(F)** Mass of tumors from NSG mice injected with parental, CD44^+^CD133^+^, or ΔCD44^+^CD133^+^ Caco-2 cells. Tumor mass in individual NSG mice was measured at 76 days after subcutaneous injection (n = 7) and is plotted as mean increases ± SD. An unpaired Student's *t*-test was used to determine statistical significance. **p < 0.01 versus control group.

To assess differences in the tumorigenic potential of CD44^+^CD133^+^ and non-CD44^+^CD133^+^ cells, Caco-2 cells were separated into CD44^+^CD133^+^-positive and CD44^+^CD133^+^-negative (ΔCD44^+^CD133^+^; a subpopulation in which the CD44^+^CD133^+^ population was depleted) fractions (Figure 3A). Unsorted Caco-2 cells (unfractionated Caco-2 cells, known as “parental cells”) were used as a control. To evaluate whether CD44^+^CD133^+^ cells were successfully isolated in the CD44^+^CD133^+^ fraction, we monitored this subpopulation by FACS analysis shortly after the first sorting. Similarly, the ΔCD44^+^CD133^+^ subpopulation was evaluated to determine whether the CD44^+^CD133^+^ component was depleted. As shown in Figure 3A, the proportion of CD44^+^CD133^+^ cells in the fractionated CD44^+^CD133^+^ subpopulation was 92.2% (middle panel), and <2% of the ΔCD44^+^CD133^+^ subpopulation were CD44^+^CD133^+^ cells (bottom panel).

Most transformed or tumorigenic cells have lost some of the dependency on growth factors that exists in untransformed or normal cells. Tumorigenic cells can proliferate in much lower serum concentrations than those required by untransformed or normal cells. To characterize the serum requirement of CD44^+^CD133^+^ Caco-2 cells, CD44^+^CD133^+^ and ∆CD44^+^CD133^+^ subpopulations were maintained for 9 days in 2% FBS. The growth rate of cells expressing CD44 and CD133 was higher than that of ∆CD44^+^CD133^+^ cells (Figure 3B), demonstrating that the need for supplemental growth factors is reduced in CD44^+^CD133^+^ cells. In addition, cell counting experiments showed that CD44^+^CD133^+^ Caco-2 cells proliferated significantly faster (3.9 times) than ∆CD44^+^CD133^+^ cells at a low (2%) serum concentration (Figure 3C).

Next, the tumor-initiating abilities of a subpopulation from Caco-2 cells were determined. Parental Caco-2 cells, purified CD44^+^CD133^+^ cells, or ΔCD44^+^CD133^+^ cells were subcutaneously injected into NSG (NOD-*scid IL2rγ^null^*) mice (n = 7 for each cell type). When injected into mice, the tumorgenicity of CD44^+^CD133^+^ cells was higher than that of parental or ΔCD44^+^CD133^+^ Caco-2 cells (Figure 3D). Tumors formed in seven out of seven mice injected with CD44^+^CD133^+^ cells. Although the ΔCD44^+^CD133^+^ subpopulation comprises a large percentage of total Caco-2 cells (76.6%; Figure 1A), this fraction did not efficiently initiate tumor formation in NSG mice (Figure 3D).

Seventy-six days after injection, tumors were entirely excised from NSG mice, and tumor photographs were taken (Figure 3E). It is notable that tumors from the CD44^+^CD133^+^ subpopulation were larger than those from parental Caco-2 cells in all seven mice.

The mass of tumors resulting from CD44^+^CD133^+^ Caco-2 cells, weighed at 76 days after subcutaneous injection, was higher than that of tumors derived from other subpopulations (Figure 3F), demonstrating that CD44^+^CD133^+^ cells have a higher tumorigenic potential and induce tumorigenesis in NSG mice.

### Secondary CD44^+^CD133^+^ Caco-2 cells from primary xenografts are also tumorigenic

**Figure 4:**
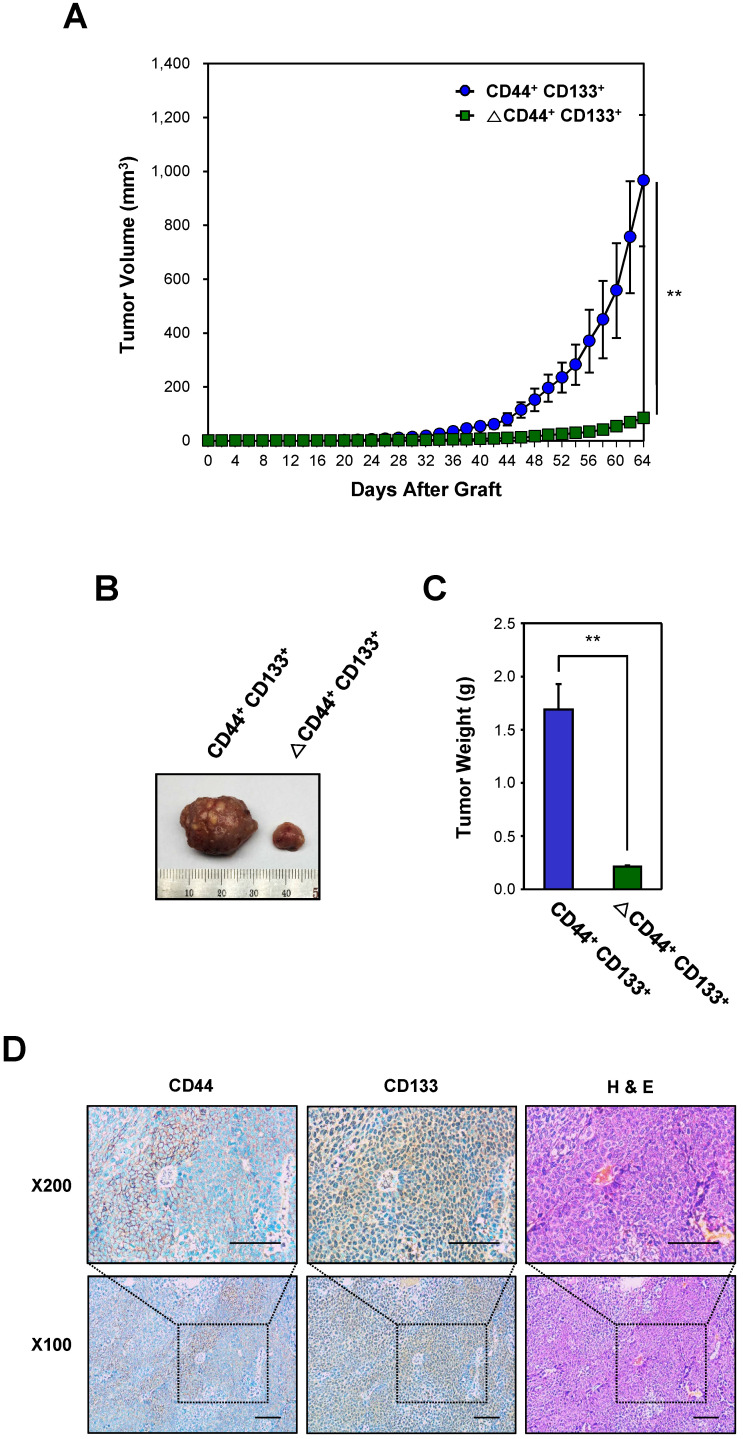
** Evaluation of the tumorigenicity of CD44^+^CD133^+^ cells from primary xenografts. (A)** Growth of mouse secondary xenografts generated after subcutaneous injection of CD44^+^CD133^+^ or ΔCD44^+^CD133^+^ cells from primary tumors. CD44^+^CD133^+^ or ΔCD44^+^CD133^+^ cells from primary xenografts were injected subcutaneously into NSG mice (n = 4). Tumor development was observed at 2 day intervals for a total of 64 days. The volume of palpable tumors derived from CD44^+^CD133^+^ (blue circles) or ΔCD44^+^CD133^+^ (green squares) cells is plotted as mean ± SD. An unpaired Student's *t*-test was used to determine statistical significance. **p < 0.01 versus control group. **(B)** Photograph of secondary xenografts generated after subcutaneous injection of CD44^+^CD133^+^ or ΔCD44^+^CD133^+^ cells from primary xenograft. Tumor photograph was taken 64 days after cell injection. **(C)** Mass of tumors from NSG mice subcutaneously injected with CD44^+^CD133^+^ or ΔCD44^+^CD133^+^ cells from primary xenografts. Tumor mass in individual NSG mice was estimated at 64 days after cell injection (n = 4) and is displayed as mean ± SD. An unpaired Student's *t*-test was used to determine statistical significance. **p < 0.01 versus control group. **(D)** Expression of CD44 and CD133 in secondary xenograft. Immunohistochemical staining of secondary xenograft demonstrated that CD44 and CD133 expression was preserved during secondary tumor development. Images are 100× (bottom panels) or 200× (top panels) magnification. Scale bars indicate 100 μm.

To investigate whether CD44^+^CD133^+^ subpopulation-derived primary tumors still harbor CD44^+^CD133^+^ Caco-2 cells with similar tumor-initiating ability, primary tumors isolated from xeno-transplanted NSG mice were excised and enzymatically dissociated. Single cells were separated into CD44^+^CD133^+^ and ΔCD44^+^CD133^+^ fractions using FACS, and purified CD44^+^CD133^+^ cells were then injected into secondary recipient NSG mice to verify whether these subpopulations were still capable of initiating tumor formation. Tumor growth was evaluated at the time of tumor appearance. As shown in Figure 4A, the CD44^+^CD133^+^ subpopulation of primary tumor-derived cells was able to initiate tumor growth *in vivo*. Interestingly the tumor growth of CD44^+^CD133^+^ cells was faster in secondary xenografts than in primary transplants in Figure 3.

A representative tumor at 64 days after subcutaneous injection of CD44^+^CD133^+^ or ΔCD44^+^CD133^+^ into NSG mice is shown in Figure 4B. The total tumor mass from individual NSG mice (at 64 days after injection) is presented in Figure 4C.

Finally, we investigated whether xenografted tumors recapitulated the histological features of primary tumors (Figure 4D). H&E staining showed that the histology and degree of differentiation of each xenografted tumor were phenotypically similar to those of the primary tumors from which it was derived. The secondary xenografted tumors were positive for CD44 and/or CD133, which mirrors the pattern seen in primary xenografts, suggesting that tumors derived from the secondary transplant of CD44^+^CD133^+^ cells into NSG mice retained similar phenotypic patterns to the primary tumor.

### Over-activation of Wnt/β-catenin signaling in the CD44^+^CD133^+^ subpopulation of Caco-2 cells

**Figure 5:**
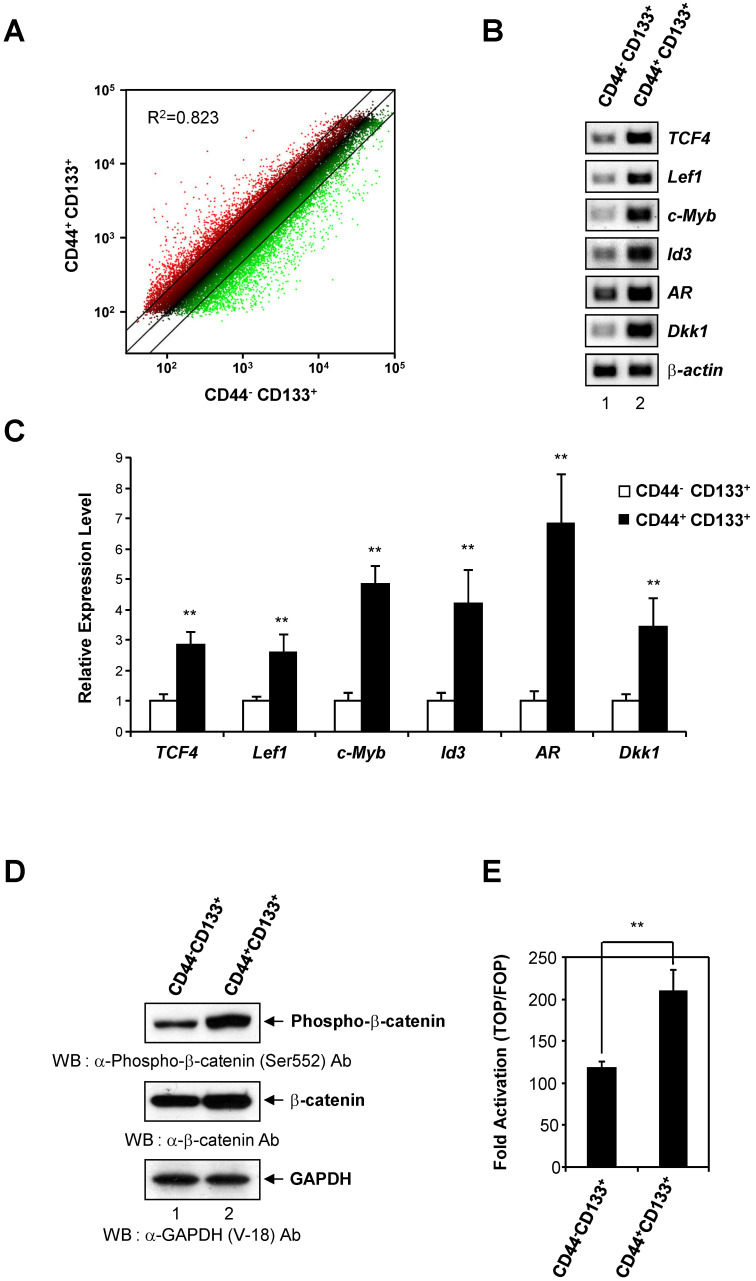
** Activation of the Wnt/β-catenin signaling pathway in CD44^+^CD133^+^ Caco-2 cells. (A)** CD44^-^CD133^+^ and CD44^+^CD133^+^ Caco-2 cells have distinct gene expression profiles. Scatter plot analysis of the gene expression pattern in CD44^+^CD133^+^ and CD44^-^CD133^+^ cells revealed distinct gene expression profiles between two subpopulations. Two-fold differences are defined above and below the lines parallel to the diagonal line. **(B)** RT-PCR analysis of genes involved in the canonical Wnt/β-catenin pathway in CD44^-^CD133^+^ and CD44^+^CD133^+^ Caco-2 cells. RT-PCR analysis of *TCF4*, *Lef1*, c-*Myb*, *Id3*, *AR*, and *Dkk1* mRNA expression was performed in CD44^-^CD133^+^ and CD44^+^CD133^+^ Caco-2 cells. *β-actin* was used for normalization. Following RT-PCR, each product was analyzed on agarose gels and stained with ethidium bromide. **(C)** TaqMan qPCR of Wnt/β-catenin pathway genes. Quantitative real-time PCR reactions were performed to analyze the relative expression of *TCF4*, *Lef1*, c-*Myb*, *Id3*, *AR*, and *Dkk1* in CD44^-^CD133^+^ and CD44^+^CD133^+^ cells, and normalized to that of *β-actin*. Representative mean ± SD from an assay performed in triplicate; two additional experiments gave similar results. An unpaired Student's *t*-test was used to determine statistical significance. **p < 0.01 versus control group. **(D)** *β-catenin* activation in CD44^+^CD133^+^ Caco-2 cells. Total cell extracts were prepared from CD44^-^CD133^+^ and CD44^+^CD133^+^ cells. The lysates were separated by SDS-PAGE and transferred to a PVDF membrane. The membrane was immunoblotted with anti-phospho-β-catenin (Ser552; Cell Signaling Technology), anti-β-catenin (BD Transduction Laboratories), or anti-GAPDH (V-18; Santa Cruz Biotechnology) antibodies. Western blot demonstrates higher levels of phospho-β-catenin (Ser552) in CD44^+^CD133^+^ cells than in CD44^-^CD133^+^ cells. **(E)** Transcriptional activation of the TOP reporter in CD44^+^CD133^+^ Caco-2 cells. The reporter plasmids, pTOPFlash (TOP, a wild-type reporter) or pFOPFlash (FOP, a mutant reporter), were transfected into CD44^-^CD133^+^ or CD44^+^CD133^+^ cells, respectively (along with a *Renilla* luciferase reporter to enable correction for transfection efficiencies), and the luciferase activity was determined. Fold induction (TOP/FOP) is expressed relative to that of the empty expression vector. Data represent mean ± SD for at least three experiments. An unpaired Student's *t*-test was used to determine statistical significance. **p < 0.01 versus control group.

To determine whether there is differential gene expression in Caco-2 cell subpopulations, genome-wide analysis of gene expression was conducted in CD44^-^CD133^+^ and CD44^+^CD133^+^ cells. The data showed a 2.0-fold or greater change in the gene expression levels between CD44^-^CD133^+^ and CD44^+^CD133^+^ subpopulations of Caco-2 cells. Scatter plot analysis showed distinct gene expression profiles (R^2^ = 0.823), suggesting that it might be possible to recognize CD44^-^CD133^+^ and CD44^+^CD133^+^ Caco-2 cells according to their gene expression patterns (Figure 5A).

The Wnt/β-catenin pathway is a critical regulatory pathway in normal colon development 28, and β-catenin is hyperactivated in colorectal cancers 28, 43. To gain insight into the role of the Wnt/β-catenin pathway in the proliferation and tumor-initiating potential of CD44^+^CD133^+^ cells, we analyzed the gene expression profile of Wnt/β-catenin pathway genes in CD44^-^CD133^+^ and CD44^+^CD133^+^ Caco-2 cells. There was a significant upregulation of Wnt/β-catenin signaling pathway genes in CD44^+^CD133^+^ cells compared with CD44^-^CD133^+^ cells. This finding was confirmed by an analysis of mRNA levels of Wnt/β-catenin signaling-related genes, such as *TCF4*, *Lef1*, *c-Myb*, *Id3*, *AR*, and *Dkk1*, by RT-PCR, which showed that the amount of transcripts encoding these genes was significantly higher in CD44^+^CD133^+^ cells than in CD44^-^CD133^+^ cells (Figure 5B). Direct sequencing of the RT-PCR products also confirmed the presence of *TCF4*, *Lef1*, *c-Myb*, *Id3*, *AR*, or *Dkk1* cDNA (data not shown).

Additionally, the mRNA levels of the Wnt/β-catenin pathway genes *TCF4*, *Lef1*, *c-Myb*, *Id3*, *AR*, and *Dkk1*, quantified using quantitative real-time PCR, were about 2.5- to 7-fold higher in CD44^+^CD133^+^ than in CD44^-^CD133^+^ cells. This result suggests that Wnt signaling is over-activated in CD44^+^CD133^+^ cells (Figure 5C).

To explore whether there was an increase in activated β-catenin protein levels, CD44^+^CD133^+^ Caco-2 cells were analyzed for the expression of phospho-β-catenin (Ser552), which is associated with transcriptional activation of downstream effectors of the canonical Wnt/β-catenin pathway 44, 45. As shown in Figure 5D (middle panel), CD44^+^CD133^+^ Caco-2 cells showed slightly higher levels of total β-catenin expression than CD44^-^CD133^+^ Caco-2 cells. Phosphorylation at serine 552 of β-catenin increases its transcriptional activity by inducing β-catenin accumulation in the nucleus 44-46. Therefore, we tested whether elevated β-catenin levels resulted in increased phosphor-β-catenin (pSer552) expression. Figure 5D (top panel) shows that the level of phospho-β-catenin (Ser552) was significantly higher in CD44^+^CD133^+^ cells, suggesting over-activation of the canonical Wnt/β-catenin pathway.

To test whether elevated phosphor-β-catenin (pSer552) levels increased transcription, CD44^-^CD133^+^ cells and CD44^+^CD133^+^ cells were each transfected with either a pTOPFlash reporter plasmid (a β-catenin responsive reporter plasmid that contains TCF binding sites 47) or a pFOPFlash mutant plasmid (a negative control counterpart that contains mutant, inactive TCF binding sites 47). There was a 1.8-fold increase in TOPFlash luciferase activity in CD44^+^CD133^+^ cells, indicating increased levels of transcriptionally active β-catenin (Figure 5E). Our results demonstrate that Wnt/β-catenin signaling is over-activated in the CD44^+^CD133^+^ subpopulation of Caco-2 cells, and suggest that this pathway is important for the proliferation and tumor-initiating properties of CD44^+^CD133^+^ Caco-2 cells.

### The Wnt/β-catenin signaling inhibitors XAV939 and IRW-1 suppress CD44^+^CD133^+^ Caco-2 cell growth *ex vivo*

**Figure 6:**
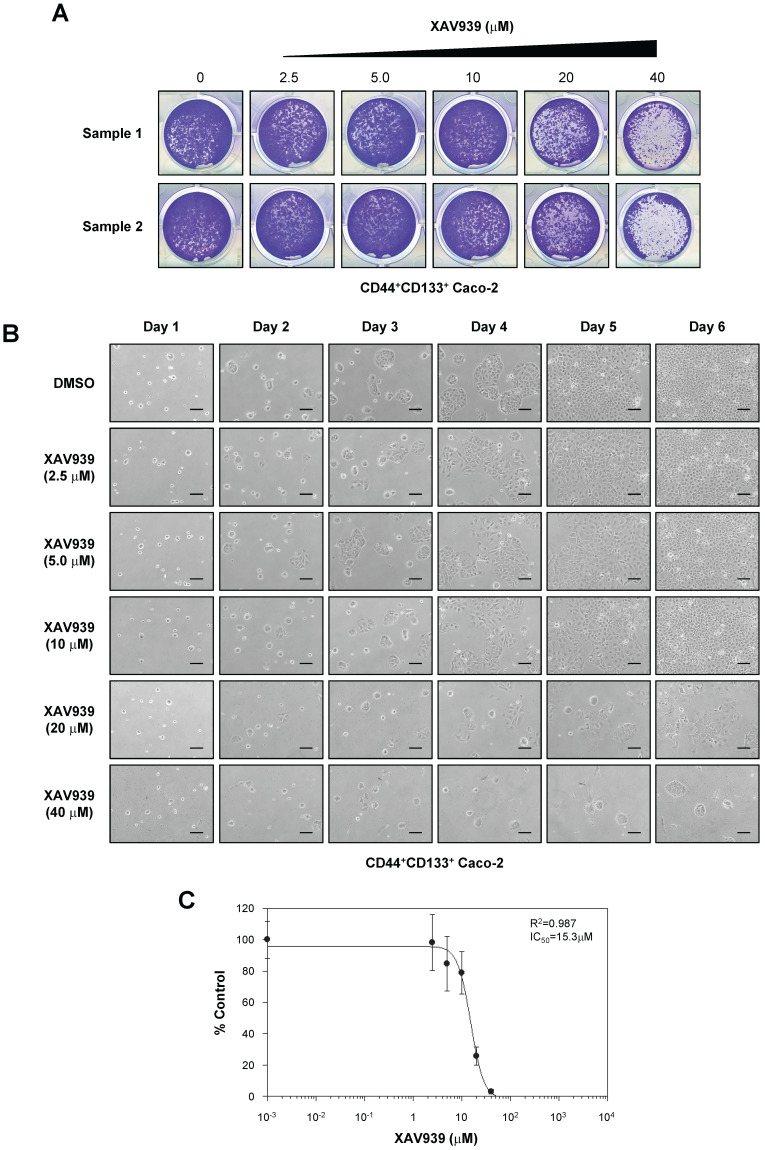
** Importance of the Wnt signaling pathway in CD44^+^CD133^+^ Caco-2 cell proliferation. (A)** Effects of XAV939 treatment on CD44^+^CD133^+^ Caco-2 cell colony formation. FACS-isolated CD44^+^CD133^+^ cells were plated in a 12-well plate at a cell density of 1 × 10^4^ per well and cultured in medium containing the indicated concentration of XAV939. Six days after XAV939 treatment, colony formation assays were carried out, and colonies were stained using crystal violet (0.05%) solution. A representative image of three separate experiments is shown. **(B)** Time-dependent effects of XAV939 treatment on CD44^+^CD133^+^ Caco-2 cell growth and morphology. CD44^+^CD133^+^ cells were cultured for 6 days in the absence or presence of the indicated concentration of XAV939. Cells were monitored once each day using an inverted phase-contrast microscope for 6 days. Scale bars indicate 100 μm. Four separate experiments produced similar results. **(C)** Determination of half-maximal inhibitory concentration of XAV939 in CD44^+^CD133^+^ Caco-2 tumor-initiating cells. Increasing concentrations of XAV939 were added to CD44^+^CD133^+^ cells, and growth inhibition was monitored by cell counting. Cell growth is presented as a percentage of the control (DMSO) at a given concentration of XAV939. Data are presented as the mean ± SD of four separate experiments. The IC_50_ value of XAV939 was calculated from a sigmoidal concentration-response curve fitted using SoftMax Pro software.

To analyze the functional significance of Wnt/β-catenin signaling in CD44^+^CD133^+^ Caco-2 cells, we analyzed the effect of XAV939, a small-molecule inhibitor of the Wnt/β-catenin signaling pathway 48, on the tumorigenic properties of CD44^+^CD133^+^ cells. As demonstrated in Figure 6A, XAV939 concentration-dependently inhibited the colony-forming ability of CD44^+^CD133^+^ Caco-2 cells.

The growth of CD44^+^CD133^+^ cells after XAV939 treatment was also examined by microscopy. XAV939 significantly inhibited the proliferation of CD44^+^CD133^+^ Caco-2 cells at all concentrations tested (Figure 6B). The IC_50_ value of XAV939 for inhibiting cell growth was 15.3 µM (Figure 6C).

**Figure 7:**
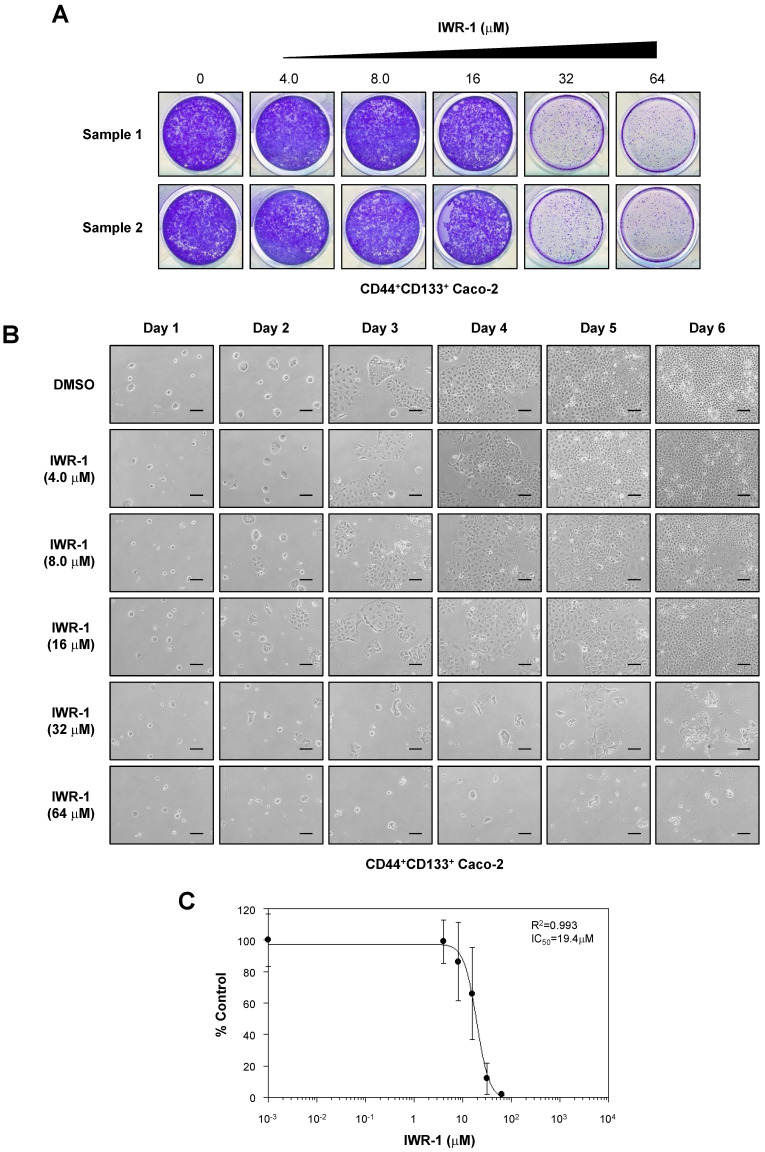
** IWR-1 inhibits proliferation of CD44^+^CD133^+^ Caco-2 cells. (A)** IWR-1 suppresses colony formation in CD44^+^CD133^+^ Caco-2 cells. CD44^+^CD133^+^ Caco-2 cells were plated in a 12-well plate at a density of 1 × 10^4^ per well and cultured in medium containing the indicated concentration of IWR-1. Six days after IWR-1 treatment, colony formation assays were carried out, and colonies were stained with 0.05% crystal violet. Representative crystal violet-stained images are presented. Three separate experiments produced similar results. **(B)** Effects of IWR-1 on CD44^+^CD133^+^ Caco-2 cell proliferation. CD44^+^CD133^+^ cells were exposed to increasing concentrations of IWR-1, and growth inhibition was monitored every day for 6 days. Scale bars indicate 100 μm. Representative images are shown from four separate experiments, which yielded similar results. **(C)** Determination of half-maximal inhibitory concentration of IWR-1. Increasing concentrations of IWR-1 were added to CD44^+^CD133^+^ cells, and growth inhibition was monitored by cell counting. Cell growth is presented as the percentage of the control (DMSO) value at a given concentration of IWR-1. Data are presented as the mean ± SD of four separate experiments. The IC_50_ value for IWR-1 was calculated from a sigmoidal concentration-response curve fitted using SoftMax Pro software.

We also assessed the anti-proliferative effects of another Wnt/β-catenin signaling inhibitor, IWR-1 49, on the proliferation of CD44^+^CD133^+^ Caco-2 cells. CD44^+^CD133^+^ Caco-2 cells treated with IWR-1 formed fewer colonies than control (DMSO)-treated cells (Figure 7A). Additionally, IWR-1 inhibited CD44^+^CD133^+^ Caco-2 cell growth in a concentration-dependent manner (Figure 7B), with an IC_50_ value of 19.4 µM (Figure 7C).

### XAV939 reduces CD44^+^CD133^+^-mediated tumor formation *in vivo*

**Figure 8:**
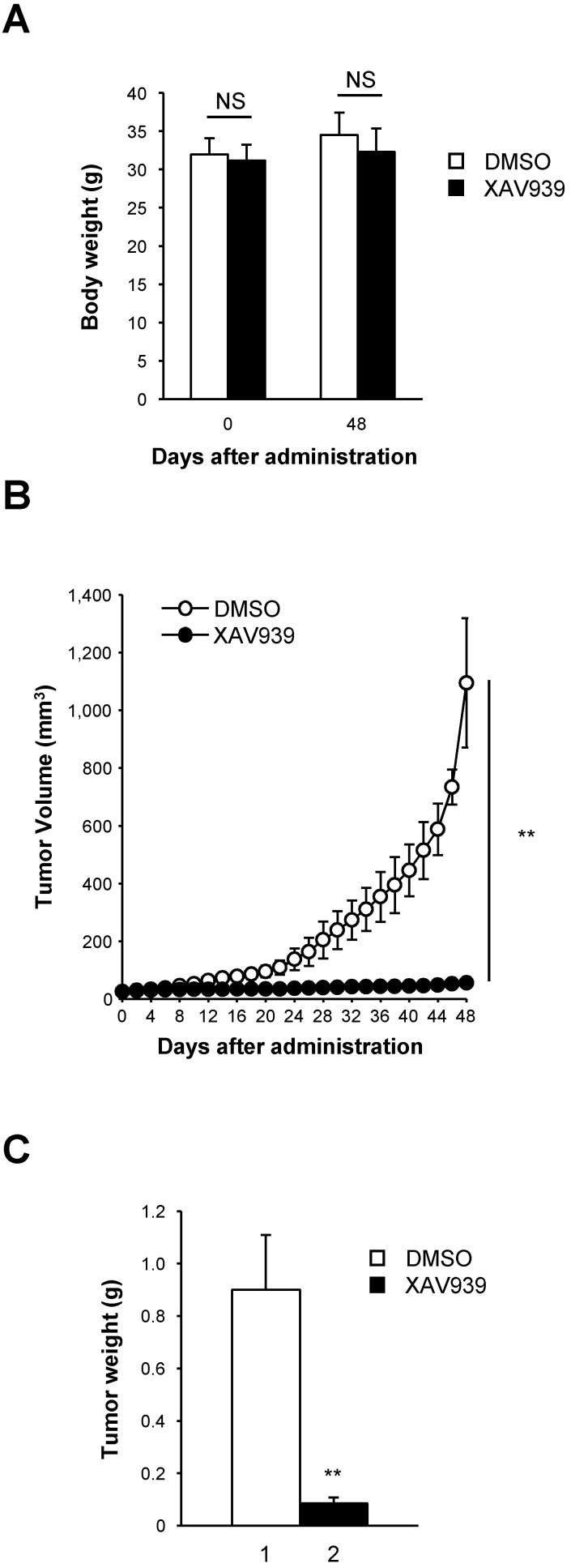
** XAV939 suppresses CD44^+^CD133^+^ cell-induced tumor formation. (A)** Mean body weight of NSG mice following XAV939 treatment. To evaluate the potential toxicity of XAV939, the body weight of NSG mice was monitored for the duration of the experiment. Mice showed no notable weight loss. An unpaired Student's *t*-test was applied to determine statistical significance. *p* = non-significant (NS) for >0.05. **(B)** Decrease in tumor volume after XAV939 treatment. Tumor-bearing mice (tumor volume = 15-25 mm^3^, randomized into two groups) were intraperitoneally injected with vehicle or XAV939 (20 mg/kg) once every 3 days for 48 days, and tumor size was monitored. An unpaired Student's *t*-test was applied to determine statistical significance. **p < 0.01 versus control group. **(C)** Quantification of tumor growth following XAV939 or control treatment. Tumor mass derived from vehicle- or XAV939-treated mice is presented as mean mass increases ± SD. An unpaired Student's *t*-test was applied to determine statistical significance. **p < 0.01 versus control group.

The tumor suppression effect of XAV939 on CD44^+^CD133^+^-mediated tumorigenesis was examined in a NSG mouse xenograft model. Approximately 1 × 10^5^ CD44^+^CD133^+^ cells were suspended in 100 μl serum-free MEM, mixed with the same volume of Matrigel, and were subcutaneously injected into 8-week-old NSG mice. When tumor volume reached 15-25 mm^3^, tumor-bearing NSG mice were randomized into two groups and treated with either XAV939 (20 mg/kg) or control (0.05 M lactic acid) by intraperitoneal injection once every 3 days for 48 days. The mass of each mouse was measured daily to ascertain general health and potential adverse effects of XAV939. There was no significant difference in the average body weight of NSG mice treated with XAV939 compared with control mice, indicating that XAV939 had no significant adverse effects. The results at day 0 (the first day of XAV939 administration) and day 48 (the last day of the experiment) are shown in Figure 8A.

Consistent with *ex vivo* experimental findings presented in Figures 6 and 7, XAV939 significantly reduced tumor volume in NSG mice compared with vehicle-treated mice (Figure 8B). At the end of treatment, XAV939 reduced tumor volume by 95% (vehicle-treated mice median tumor volume = 1095.1 ± 224.2 mm^3^; XAV939-treated mice median tumor volume = 55.2 ± 8.5 mm^3^; n = 9, *p* < 0.01). These results indicate that supressing Wnt/β-catenin signaling impairs CD44^+^CD133^+^-induced tumor growth *in vivo*.

There was a significant reduction in tumor mass in mice treated with XAV939 (Figure 8C). On the last day (48 days after XAV939 treatment initiation) of the experiment, the mean tumor mass was 0.90 ± 0.21 g in vehicle-treated animals and 0.09 ± 0.02 g in XAV939-injected NSG mice (Figure 8C), representing a reduction in tumor mass of 82.2% (*p* < 0.01). These results indicate that the elevated expression of Wnt/β-catenin signaling genes is associated with the tumorigenic potential of CD44^+^CD133^+^ Caco-2 cells. Thus, our data indicate that Wnt/β-catenin signaling is essential for CD44^+^CD133^+^ cell-mediated tumorigenesis *in vivo*, and that XAV939 inhibits the tumorigenic potential of CD44^+^CD133^+^ Caco-2 cells without apparent toxic side effects.

## Discussion

Many studies have attempted to identify the biological parameters that endow cancer cells with increased aggressiveness, independently of the known prognostic clinic-pathological features of colorectal cancer. Despite earlier studies demonstrating that human colorectal tumor-initiating cells are enriched for CD44 or/and CD133 marker proteins 26-28, little is known about how these populations contribute to tumorigenesis. To elucidate the pathways that regulate tumor-initiating Caco-2 cell growth and survival, we characterized the biological properties of CD44^-^CD133^-^, CD44^-^CD133^+^, and CD44^+^CD133^+^ subpopulations using *in vitro* and *in vivo* systems. To investigate tumorigenic potential, we divided Caco-2 cells into two groups, namely, CD44^+^CD133^+^ and non-CD44^+^CD133^+^ cells, because the growth properties of CD44^+^CD133^+^ Caco-2 cells markedly differed from those of CD44^-^CD133^+^ and CD44^-^CD133^-^ Caco-2 cells. Additionally, to determine whether there was differential gene expression in Caco-2 cell subpopulations, gene expression was analyzed in CD44^-^CD133^+^ and CD44^+^CD133^+^ cells because these cells formed successive colonies and grew similar to normal unfractionated cells, whereas CD44^-^CD133^-^ cells failed to form colonies. We ultimately found that only CD44^+^CD133^+^ cells could form new tumors.

Genes previously associated with normal intestinal stem cells were upregulated in the CD44^+^CD133^+^ subpopulation of Caco-2 cells. Wnt/β-catenin signaling is one of the critical cascades regulating the development and stemness of intestinal stem cells 50, 51. Aberrant regulation of Wnt/β-catenin signaling is a common theme seen across many human cancer types 52, 53. Consistent with this theme, our results showed that the expression of genes involved in the Wnt/β-catenin pathway, such as *TCF4*, *Lef1*, *c-Myb*, *Id3*, *AR*, and *Dkk1*, is over-activated in CD44^+^CD133^+^ Caco-2 cells. The level of active β-catenin also increased significantly in CD44^+^CD133^+^ Caco-2 cells, indicating that upregulation of the canonical Wnt/β-catenin pathway may be essential for the growth and tumorigenicity of CD44^+^CD133^+^ Caco-2 cells *in vitro* and *in vivo*. Although a direct role for Wnt/β-catenin pathway upregulation in maintaining the tumor-initiating properties of CD44^+^CD133^+^ Caco-2 cells needs to be confirmed, our results suggest that this is plausible.

According to our data, CD44^+^CD133^+^ cells were more mitotically active than CD44^-^CD133^-^ and CD44^-^CD133^+^ cells, and only the CD44^+^CD133^+^ subpopulation of Caco-2 cells was endowed with tumorigenic potential when transplanted into NSG mice. Because conventional chemotherapeutic strategies are unable to completely eradicate tumor-initiating cells, it is essential to investigate potential therapies that target tumor-initiating cells in human colorectal cancer 54, 55. We observed novel tumor-initiating cell-like characteristics in a CD44^+^CD133^+^ subpopulation of Caco-2 cells, and showed that treating this subpopulation with XAV939 repressed tumor-initiating properties, including suppression of cell proliferation in culture and tumorigenic potential in mice. XAV939 is a tankyrase inhibitor 48. Tankyrase inhibition induces the antiproliferative effect of the Axin-GSK3β complex, which negatively affects Wnt/β-catenin signaling. We have provided evidence that small-molecule inhibitors of the Wnt/β-catenin signaling pathway might be used therapeutically to specifically target the tumor-initiating cell population in colorectal cancer. Although it seems reasonable to use Wnt/β-catenin inhibitors as potent chemotherapeutic drugs targeting colorectal tumor-initiating cells, therapeutic targeting of the Wnt/β-catenin signaling pathway is the most challenging task for treatment of human cancer. Unfortunately, targeting Wnt/β-catenin signaling molecules causes serious side effects because Wnt/β-catenin signal transduction is also necessary for the biological function of intestinal stem cells 56. Therefore, clinical trials of Wnt/β-catenin inhibitors as chemotherapeutic drugs are likely restricted by cytotoxicity. Better understanding of CD44^+^CD133^+^ tumor-initiating cells will help to overcome this problem and improve therapeutic strategies.

Our results also demonstrated that tumor growth caused by CD44^+^CD133^+^ tumor-initiating Caco-2 cells separated from the primary tumor was faster than that in primary xenografts. The enhanced ability of CD44^+^CD133^+^ tumor-initiating cells of primary xenografts to initiate tumor formation may be related to the abundance of CD44^+^CD133^+^ tumor-initiating cells in secondary xenografts after injection of NSG mice or Wnt/β-catenin signaling may be further activated in CD44^+^CD133^+^ cells in secondary xenografts. Additionally, it is possible that CD44^+^CD133^+^ tumor-initiating cells from primary xenografts have an enhanced ability to respond to tumor microenvironmental signals via dynamic interactions with their microenvironment in secondary xenografts. However, the reason for the observed increased tumorigenic potential of CD44^+^CD133^+^ tumor-initiating cells in primary xenografts is largely unknown and a mechanistic explanation for the enhanced tumorigenicity of the CD44^+^CD133^+^ subpopulation of primary xenografts remains enigmatic. We are currently investigating how and why CD44^+^CD133^+^ tumor-initiating Caco-2 cells isolated from primary xenografts form tumors faster than parental Caco-2 cells. The identification of additional genes that maintain and regulate the tumor-initiating properties of CD44^+^CD133^+^ Caco-2 cells might provide rational targets for therapeutic intervention.

## Conclusions

The results of this study demonstrate that CD44^+^CD133^+^ Caco-2 cells have characteristics of tumor-initiating cells. They have altered cell cycle kinetics and gene expression profiles. These increased growth characteristics and the ability to self-renew are partially due to preferential activation of the Wnt/β-catenin signaling pathway. These findings increase our understanding of the biological characteristics of CD44^+^CD133^+^ Caco-2 cells. Notably, primary tumors grown from CD44^+^CD133^+^ Caco-2 cells contain a mixed population of both CD44^+^CD133^+^ and non-CD44^+^CD133^+^ subpopulations, indicating that CD44^+^CD133^+^ cells are capable of re-creating the full phenotypic heterogeneity of the parent Caco-2 cell line. Interestingly the tumor growth of CD44^+^CD133^+^ was faster in secondary xenografts than in primary transplants. These data suggest that the CD44^+^CD133^+^ subpopulation of Caco-2 cells should be a target for the design of new therapeutic strategies. Further characterization of the phenotypic and genetic features of this subpopulation could suggest novel therapeutic directions.

## Supplementary Material

Supplementary table S1.

## Figures and Tables

**Table 1:** Expression of CD44 and CD133 proteins in colorectal cancer cell lines

Cell lines	CD44 (%)*	CD133 (%)*
Caco-2	23.4	98.4
HCT116	81.1	83.0
HT29	88.0	78.1
SW480	63.2	8.3
DLD1	63.1	12.3

*Results represented average of three individual flow cytometry experiments.

## Abbreviations

CD: cluster of differentiation
PCR: polymerase chain reaction
RT-PCR: reverse transcriptase-polymerase chain reaction
FACS: fluorescence-activated cell sorting
WB: western blot
DMSO: dimethyl sulfoxide
cDNA: complementary DNA
