# Supplementary material for: Wnt/β-catenin Signaling Inhibitors suppress the Tumor-initiating properties of a CD44+CD133+ subpopulation of Caco-2 cells
Source: Int J Biol Sci. 2021 Apr 12;17(7):1644–59. doi: 10.7150/ijbs.58612 (PMC8120464; doi:10.7150/ijbs.58612)
Supplement: Supplementary file 1 — Supplementary table S1. [file ijbsv17p1644s1.pdf]

Supplementary Table 1

Primers used for RT-PCR and Quantitative RT-PCR

**Name**	**Sequence**
TCF4 F [1]	5'-CGAGGGTGATGAGAACCTGC-3'
TCF4 R [1]	5'-CCCATGTGATTCGATGCGT-3'
Lef1 F [2]	5'-CCAGCTATTGTAACACCTCA-3'
Lef1 R [2]	5'-TCAGATGTAGGCAGCTGTCA-3'
c-Myb F [3]	5'-CCGATGGCAGAAAGTACT-3'
c-Myb R [3]	5'-CTGCCCATCTGTTCCCCA-3'
Id3 F [4]	5'-AACTTCGCCCTGCCCACTTG-3'
Id3 R [4]	5'-CACCTCCACGCTCTGAAAAG-3'
AR F [5]	5'-CTCTCTCAAGAGTTTGGA-3'
AR R [5]	5'-CACTTGCACAGAGATGAT-3'
Dkk1 F [6]	5'-TCCGAGGAGAAATTGAGG-3'
Dkk1 R [6]	5'-CCTGAGGCACAGTCTGAT-3'

Supplementary References

1. Yang LH, Xu HT, Han Y, Li QC, Liu Y, Zhao Y, et al. Axin downregulates TCF-4 transcription via beta-catenin, but not p53, and inhibits the proliferation and invasion of lung cancer cells. Mol Cancer. 2010; 9: 25.
2. Zhao DH, Hong JJ, Guo SY, Yang RL, Yuan J, Wen CY, et al. Aberrant expression and function of TCF4 in the proliferation of hepatocellular carcinoma cell line BEL-7402. Cell Res. 2004; 14: 74-80.
3. Xu H, Inouye M, Hines ER, Collins JF, Ghishan FK. Transcriptional regulation of the human NaPi-IIb cotransporter by EGF in Caco-2 cells involves c-myb. Am J Physiol Cell Physiol. 2003; 284: C1262-71.
4. Obayashi S, Tabunoki H, Kim SU, Satoh J. Gene expression profiling of human neural progenitor cells following the serum-induced astrocyte differentiation. Cell Mol Neurobiol. 2009; 29: 423-38.
5. Lau KM, Mok SC, Ho SM. Expression of human estrogen receptor-alpha and -beta, progesterone receptor, and androgen receptor mRNA in normal and malignant ovarian epithelial cells. Proc Natl Acad Sci U S A. 1999; 96: 5722-7.
6. Maehata T, Taniguchi H, Yamamoto H, Nosho K, Adachi Y, Miyamoto N, et al. Transcriptional silencing of Dickkopf gene family by CpG island hypermethylation in

human gastrointestinal cancer. World J Gastroenterol. 2008; 14: 2702-14.
